# Testing the Weiss-Harter-Model: Physical Activity, Self-Esteem, Enjoyment, and Social Support in Children and Adolescents

**DOI:** 10.3389/fpsyg.2019.02568

**Published:** 2019-11-15

**Authors:** Darko Jekauc, Carina Mnich, Claudia Niessner, Kathrin Wunsch, Claudio R. Nigg, Janina Krell-Roesch, Alexander Woll

**Affiliations:** Institute of Sports and Sports Science, Karlsruhe Institute of Technology, Karlsruhe, Germany

**Keywords:** physical activity, children, adolescents, self-esteem, enjoyment, perceived competence, social support, Weiss-Harter-model

## Abstract

**Background:**

Several theories have been established to explain physical activity (PA) participation. However, many of these theories might not be applicable to adolescent PA behavior as they require a high level of cognitive reflection. Weiss suggests a model for youth which is based on the theoretical concept of Harter, focusing on self-esteem within social, emotional, and developmental aspects to explain behavior. The aim of this study was to test the original and a social support focused alternative version of the Weiss-Harter-model, and to cross-validate the findings in two separate studies.

**Methods:**

Data from two cross-sectional studies was retrieved and the models tested using structural equation modeling. Participants aged 11–17 years were recruited from a school (Study 1: *N* = 182) and from the German MoMo study (Study 2: *N* = 2,274). They filled in questionnaires about perceived competence, social support, self-esteem, PA enjoyment, and minutes of moderate-vigorous PA (MVPA).

**Results:**

None of the studies showed a good model fit for the original model [Study 1: CFI = 0.870, RMSEA 0.118 (90% CI 0.081–0.158), χ*^2^* = 38.7, *p* < 0.01; Study 2: CFI = 0.871, RMSEA 0.148 (90% CI 0.140–0.155), χ*^2^* = 1112.6, *p* < 0.01], explaining only 12% and 17% of MVPA variance, respectively. The alternative model which added the direct paths of social support to MVPA and PA enjoyment had a very good model fit for both Study 1 [CFI = 1.000, RMSEA 0.000 (90% CI 0.000–0.031), χ*^2^* = 4.8, *p* > 0.05] and Study 2 [CFI = 0.990, RMSEA 0.043 (90% CI 0.035–0.051), χ*^2^* = 103.7, *p* < 0.01]. The addition of these paths led to changes in effect size and directions of other path coefficients, with self-esteem having a small to meaningless impact on MVPA. The revised models accounted for 38% and 42% explained variance in MVPA, respectively.

**Discussion:**

The prominent role of self-esteem in the original model could not be confirmed. Instead, the results emphasize the role of social support for PA and PA enjoyment, which is in line with current research. Interventions to increase adolescent PA levels should thus focus more on components of social support instead of self-esteem. Future studies are needed to examine the interplay between social support, PA enjoyment and perceived competence as PA determinants.

## Introduction

Physical activity (PA) is an important lifestyle factor to achieve or maintain health in children and adolescents. PA is related to physical health, including improved physical fitness, normal weight, bone strength, and cardiovascular health, and mental health, such as less psychological distress and less depressive symptoms (Janssen and Leblanc, 2010; Poitras et al., 2016; Miguel-Berges et al., 2018; Skrede et al., 2018) The WHO recommends 60 min of moderate to vigorous PA (MVPA) daily for children and adolescents (World Health Organization [WHO], 2010). However, globally, only about 20% of youth meet the WHO-guidelines (Sallis et al., 2016) which is similar to prevalence rates reported for Germany (Jekauc et al., 2012a; Demetriou et al., 2018). In Germany, a considerable part of adolescents PA takes place in sports clubs; however, the amount of PA also decreases during adolescence (Jekauc et al., 2013a; Schmidt et al., 2017). As physical inactivity is the fourth leading risk factor for global mortality (World Health Organization [WHO], 2018), mechanisms and determinants of PA in youth and childhood must be identified and subsequently used to design interventions that increase and maintain PA levels through all age groups to combat the global pandemic of physical inactivity.

Various theories considering psychosocial determinants have been examined to explain PA in adults, such as the social cognitive theory (Bandura, 1998) and the theory of planned behavior (Ajzen, 1991). accounting for about 30% of explained behavioral variance (Armitage and Conner, 2001; Young et al., 2014). While these theories are based on cognitive reflections which allow for conscious behavioral control in adults, the same cognitive processes determining behavior may not be applicable to developing children, and adolescents (Conner and Norman, 2005). Due to the development of the brain, adolescents differ substantially from adults in various aspects of cognitive control processes, such as response inhibition and working memory, so that cognitive control is limited (Luna et al., 2010). Instead, emotional (Jekauc and Brand, 2017) as well as developmental (Jekauc, 2009) aspects might be important determinants of PA in youth.

As an alternative to current behavior change theories, Harter developed a self-worth model based on principles of developmental psychology. The model aims to explain the motivation of 8–18 year old children and adolescent for behavior, while simultaneously considering social and emotional aspects in her model (Harter, 1987). Harter proposed the concept of global self-esteem that includes subgroups such as school-related and physical self-esteem (Harter, 1987). She combined two different approaches of global self-esteem in her model, based on prior research. Cooley (1964) considered global self-esteem as a result of social interaction with personally important people and their opinions about the behavior. In contrast, James (1984) assumed a cognitive-analytical model, that regards global self-esteem as a result of high congruence between actual success in certain domains and the personal meaning of these domains.

Harter combined both approaches as complementary concepts in her model, with social support and perceived competence predicting self-esteem (Harter, 1987). This assumption has been empirically confirmed (Harter, 1990; Miller, 2000). Beyond that, Harter assumed that self-esteem mediates the impact of perceived competence and social support on affect. Additionally, self-esteem is partially mediated through affective response on motivation. Affect has a direct effect on motivation for certain behaviors. These hypotheses found empirical support (Harter, 1990, 1999; Oosterwegel et al., 2001).

**FIGURE 1:**
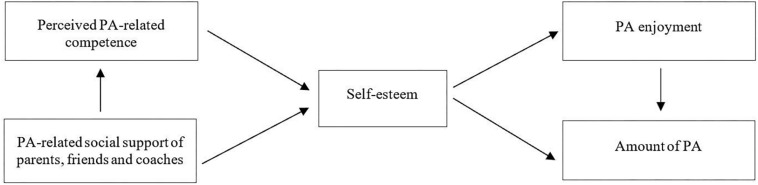
Development model of PA (Weiss, 2000).

Based on empirical findings revealing that self-perception can explain differences in sports performance, endurance as well as in positive and negative affective reactions (Weiss, 2008), Weiss applied Harter’s model to PA (Weiss, 2000; see Figure 1). She adapted and restricted the scope of the components to fit PA behavior and hypothesized that PA-related social support and perceived PA-related competence improve PA-related self-esteem. High self-esteem is considered to cause positive affect and reducing negative affect, summarized as PA enjoyment. PA enjoyment and self-esteem are hypothesized to predict the amount of PA (Ebbeck and Weiss, 1998).

The relationships of the models’ components with PA have been reported. Perceived competence describes youth’s perception about their own abilities to master certain tasks (Harter, 1985; Wienke and Jekauc, 2016), and it has been positively associated with higher levels of PA in children and adolescents (Carroll and Loumidis, 2001; Gao, 2008; Haugen et al., 2011; Inchley et al., 2011; Leisterer and Jekauc, 2019b). Additionally, perceived sports competence and perceived physical condition have been found to account for 27% variance in overall PA level (Crocker et al., 2000). Another study showed that physical self-concept is an important mediator of the relationship between motor abilities and PA in youth (Jekauc et al., 2017). In a meta-analysis examining physical self-concept and PA in youth, perceived competence was most strongly related to PA, followed by perceived fitness (Babic et al., 2014). Beyond this, higher perceived competence in school physical education showed a moderate relationship with physical education enjoyment (Carroll and Loumidis, 2001; Gao, 2008; Cairney et al., 2012; Leisterer and Jekauc, 2019b). Physical self-esteem has also been associated with higher levels of PA in children and adolescents (Crocker et al., 2000; Inchley et al., 2011), showing a small relationship with MVPA (Raudsepp et al., 2002).

Furthermore, PA enjoyment has also been associated with higher amounts of PA (Carroll and Loumidis, 2001; Jekauc, 2015; Bai et al., 2018), and accounted for 17% of explained variance in pedometer-assessed PA (Gao, 2008).

Various studies also showed the importance of social support for children’s and adolescent’s PA. Social support refers to resources that are perceived to improve the well-being of the recipient (Shumaker and Brownell, 1984). Positive associations have been reported between PA and parental support, direct parent help, and parent’s PA (Geller et al., 2013; Sterdt et al., 2013; Draper et al., 2015), and support and encouragement from significant others (Sterdt et al., 2013; Jaeschke et al., 2017). Additionally, parental and peer social support have also been shown to be connected to PA enjoyment (Silva et al., 2014; Wing et al., 2016; Shen et al., 2018).

While the relationship between the single components of the Weiss-Harter-model (2000) and PA in youth have been well explored, fewer studies have investigated the relationship between the components of the entire model. Most studies comprised variables to assess perceived PA-related competence and PA-related global self-esteem, but, to date, little is known about the integrated relationship with social support and PA enjoyment.

Therefore, the first aim of this study was (a) to test the proposed model as a whole (Weiss, 2000; see Figure 1). As the current body of research supports the role of social support for PA enjoyment and PA, an adapted version of the model with a focus on social support was also established. The second aim was (b) to test this adapted model, and the third aim was (c) to cross-validate the findings in two separate studies with independent samples.

## Materials and Methods

A cross-validation technique was used to test and further adapt the Weiss-Harter-model of PA, using data derived from two different studies. Study 1 can be regarded as a pilot study and was conducted to test the original model and to adapt the original model due to possible misspecifications of the original model. Study 2 was used to confirm both the original model and the adaptations made in Study 1.

### Study 1

#### Participants

Participants were recruited from a comprehensive school including all three types of secondary schools (Hauptschule, Realschule, and Gymnasium) in the German tripartite school system. From each school, three classes (fifth, seventh, and ninth class) were randomly recruited so that the proportion of each school type remained equal. The responsible class teachers and physical education teachers were asked about their interest in participating in this study after having the study design and purpose explained to them. After the teachers had agreed, informed written consent was obtained from the participants and their parents or guardians before entering into the study according to Helsinki Declaration. The study was approved by the ethics committee of the Charité Universitätsmedizin Berlin. Participants aged between 11 and 17 years (*N* = 187; 44.4% female; *M*age = 13.0 years; *SD* = 1.4) were recruited.

#### Measures

##### Physical activity

Participants completed the MoMo Physical Activity Questionnaire (MoMo-PAQ; Woll et al., 2011) for adolescents. Reliability and validity of this questionnaire were shown to be comparable to other international PA questionnaires for youth (Jekauc et al., 2013c). MVPA includes measures on MVPA in school, during leisure, in everyday activities and in sports clubs. The outcome measure was minutes of MVPA per week.

##### Social support

Social support was measured using a social support scale with 8 items (Reimers et al., 2012). Participants were asked how much they feel supported by family (parental social support: 5 items) and friends (peer social support: 3 items) to be physically active, using a 4-point Likert scale (1, “*never*” and 4, “*always*”). Cronbach’s alpha for peer social support was 0.70 and for parental social support 0.77. The scale showed moderate test-retest reliability, good internal consistency and good predictive validity (Reimers et al., 2012).

##### Self-esteem

General self-esteem was assessed with 4 items positive poled items, and physical self-esteem with 3 items (2 positive, 1 negative poled) which have already been used in the German Sprint-Study (Sportbund, 2006). The scale assesses the degree of acceptance of one’s own person which allows to assess the current psychological well-being of students (Sportbund, 2006), such as “*In general, I’m happy with myself.*” Physical self-esteem contributes to general self-esteem (Fox, 1990). Therefore, participant’s satisfaction with their own attractiveness was assessed (Sportbund, 2006), e.g., “*I like my body the way it is.*” The items are based on Fox’s Physical Self-Perception profile (Fox, 1990; Sportbund, 2006). Participants could score on a 4-point Likert scale (1: “*not true at all*,” 4: “*exactly true*”). Validity and reliability have been confirmed (Welk et al., 1997). Cronbach’s alpha for physical self-esteem was 0.82 and for general self-esteem 0.85.

##### Physical activity enjoyment

Physical activity enjoyment was assessed through 16 items (9 positive and 7 negative poled items) on a 5-point Likert-Scale, using the “physical activity enjoyment scale” (PACES) (Motl et al., 2001; Jekauc et al., 2013b). Validity and reliability for children and adolescents have been tested internationally (Motl et al., 2001; Carraro et al., 2008; Fernández et al., 2008; Paxton et al., 2008; Dunton et al., 2009) and for the German version (Jekauc et al., 2013b). The negative scale was reverse scored such that higher scores indicate higher PA enjoyment. PACES positive had a Cronbach’s alpha of 0.89 and PACES negative of 0.82.

##### Perceived competence

Was not assessed in Study 1 due to the length of the questionnaire and lack of time.

### Study 2

#### Participants

The second dataset is derived from the Motorik-Modul Longitudinal Study (MoMo; Wagner et al., 2013) which is an in-depth study of the German Health Interview and Examination Survey for Children and Adolescent (KIGGS; Kurth et al., 2008). A stratified, multi-stage sample with three evaluation levels was drawn (Kamtsiuris et al., 2007). First, a systematic sample of 167 primary sampling units was selected from an inventory of German communities stratified according to the classification system that measures the level of urbanization and geographic distribution. Second, an age-stratified sample of randomly selected children and adolescents was drawn from the official registers of local residents. A total of 12,368 children and adolescents participated in the KiGGS Wave 1 (Lange et al., 2014), of which 6,076 were randomly assigned to MoMo Wave 1. Thus, the MoMo Study is based on a subsample of the KiGGS Study. The first survey wave (baseline) of the MoMo Longitudinal Study was conducted between 2003 and 2006 using a nationwide representative sample (Kamtsiuris et al., 2007). The first follow-up began 6 years later in September 2009 and ended in July 2012 (Jekauc et al., 2017). For this study, only data of the follow-up were used with 2,274 participants (50.3% female) aged between 11 and 17 years (*M* = 14.4 years; *SD* = 2.0) as this was the only wave that included all variables required to test the model in the questionnaires. Results, design and sampling procedure of the MoMo Baseline and Longitudinal Study have been previously published (Kamtsiuris et al., 2007; Wagner et al., 2013). Ethical approval was obtained by the Charité Universitätsmedizin Berlin ethics committee and the Federal Office for the Protection of Data. The study was conducted according to the Declaration of Helsinki. For both the MoMo- and the KIGGS-study, participants gave their written consent to participate and were informed in detail about the study and data management by the Robert Koch Institute. Parents gave their written consent for minors and the presence of a legal guardian was mandatory for participants under the age of 15.

#### Measures

##### Physical activity, physical activity enjoyment, social support, and self-esteem

The same measures as in Study 1 were used.

##### Perceived competence

Perceived competence was measured using the questionnaire for the assessment of the physical self-concept (Stiller et al., 2004). The questionnaire is widely related to the Physical Self-Description Questionnaire (Marsh and Redmayne, 1994). The questionnaire consists of 46 items that are divided into seven subscales measuring perceived motor skills (36 items: strength, flexibility, endurance, speed, coordination, and general sports competence) and perceived physical attractiveness (10 items). Participants could respond on a 4-point Likert scale (1, “*does not apply*” and 4, “*does apply*”). Reliability and validity of the questionnaire for children in adolescents could be shown (Stiller et al., 2004). In the MoMo-study, these questions were only asked to children and adolescents aged 11 years and older (Schmidt et al., 2016). General sports competence was treated as one subscale; strength, flexibility, endurance, speed, and coordination were summarized to another subscale by calculating the mean of the five skills. As physical attractiveness does not reflect competence, this scale was not included in the analyses. The scale has a good internal consistency, as indicated by Cronbach’s Alpha of 0.94 for motor skills and 0.90 for general sports competence.

### Statistical Analysis

Structural equation modeling (SEM) was used to test the model proposed by Weiss (2000). SEM allows to test how constructs are defined through variables and how these constructs are inter-related. In this way, it can be shown how the sample data fits the theoretical model (Schumacker and Lomax, 2011). The models in Study 1 and Study 2 differed slightly because perceived competence was not included for the model in Study 1.

To assess the overall model fit, χ*^2^*-statistic was used. A good model-fit is indicated with an insignificant p value (Barrett, 2007). However, the χ*^2^*-statistic is sample size dependent (Hu and Bentler, 1999). The comparative fit index (CFI) shows the relative improvement in fit by comparing the baseline model with the suggested model. CFI values around 0.95 indicate a good fit, values around 0.90 indicate an acceptable fit (Hu and Bentler, 1999). The root-mean-square error of approximation (RMSEA) describes the error of approximation in the population, showing the model’s closeness of fit. RMSEA of ≤0.06 indicate a good model fit (Hu and Bentler, 1999; Hooper et al., 2008). The successive, nested models were tested by χ*^2^*-difference tests. To handle missing data, full-information maximum likelihood estimation was performed using AMOS 25. Through this method, less biased estimates are obtained than through classical missing data procedures, including list-/pairwise deletion or mean imputation (Jekauc et al., 2012b). For model testing purposes, items were organized in subscales. Taking items together has already been done previously to simplify model testing as this improves reliability and decreases idiosyncratic variance (Nigg et al., 2001).

## Results

### Descriptives

In Study 1, 155 participants out of 182 had complete datasets (15% missing data). In Study 2, 1,849 participants out of 2,274 had complete data sets (19% missing data). The means, standard deviations and confidence intervals for means of the both studies are presented in Table 1.

**TABLE 1:** Descriptives of age and study constructs.

	**Study 1 (*N* = 182)**		**Study 2 (*N* = 2,274)**	
	**Mean (*SD*)**	**95%-CI**	**Mean (*SD*)**	**95%-CI**
Age	13.03 (1.43)	12.82–13.24	14.38 (2.00)	14.30–14.46
**Physical self-concept**				
Motor skills		17.78 (2.90)	17.65–17.91	
General sports competence		18.28 (1.74)	18.21–18.35	
**Social support**				
Peer social support	2.84 (0.55)	2.76–2.92	2.57 (0.61)	2.54–2.60
Parental social support	2.74 (0.61)	2.65–2.83	2.86 (0.60)	2.83–2.89
**Self-esteem**				
Physical self-esteem	9.55 (2.23)	9.22–9.88	9.26 (2.07)	9.17–9.35
General self-esteem	13.13 (2.42)	12.77–13.48	12.99 (2.34)	12.89–13.09
**Enjoyment**				
PACES positive	3.77 (0.79)	3.65–3.89	3.83 (0.74)	3.80–3.86
PACES negative	4.58 (0.56)	4.50–4.66	4.51 (0.58)	4.49–4.53
Weekly MVPA minutes	315.69 (270.51)	276.13–355.25	302.00 (218.75)	293.01–310.99

The correlations between variables of interest for study 1 are presented in Table 2, and for study 2 in Table 3. Both studies show a comparable pattern of correlations that are consistently in the conceptual direction and with correlations ranging from small to large. In Study 1, PA was significantly correlated with parental social support as well as positive and negative PACES. Physical and general self-esteem as well as peer social support were not significantly correlated with PA. In Study 2, all predictors were significantly correlated with PA.

**TABLE 2:** Correlation table Study 1.

	**Parental social support**	**Physical self-esteem**	**General self-esteem**	**PACES positive**	**PACES negative**	**MVPA minutes**
Peer social support	0.172^∗^	0.106	0.115	0.225^∗∗^	0.132	0.143
Parental social support	–	0.215^∗∗^	0.234^∗∗^	0.294^∗∗^	0.246^∗∗^	0.349^∗∗^
Physical self-esteem		–	0.690^∗∗^	0.011	0.018	0.035
General self-esteem			–	0.103	0.072	0.102
PACES positive				–	0.521^∗∗^	0.291^∗∗^
PACES negative					–	0.203^∗∗^

*^∗^*p* < 0.05 and ^∗∗^*p* < 0.01.*

**TABLE 3:** Correlation table Study 2.

	**SC general sports competence**	**Peer social support**	**Parental social support**	**Physical self-esteem**	**General self-esteem**	**PACES positive**	**PACES negative**	**MVPA minutes**
SC motor skills	0.810^∗∗^	0.343^∗^	0.427^∗∗^	0.465^∗∗^	0.473^∗∗^	0.576^∗∗^	0.491^∗∗^	0.393^∗∗^
SC general sports competence	–	0.390^∗∗^	0.438^∗∗^	0.386^∗∗^	0.392^∗∗^	0.546^∗∗^	0.467^∗∗^	0.388^∗∗^
Peer social support		–	0.355^∗∗^	0.157^∗∗^	0.188^∗∗^	0.394^∗∗^	0.344^∗∗^	0.319^∗∗^
Parental social support			–	0.258^∗∗^	0.276^∗∗^	0.426^∗∗^	0.363^∗∗^	0.364^∗∗^
Physical self-esteem				–	0.722^∗∗^	0.299^∗∗^	0.303^∗∗^	0.142^∗∗^
General self-esteem					–	0.330^∗∗^	0.297^∗∗^	0.160^∗∗^
PACES positive						–	0.691^∗∗^	0.371^∗∗^
PACES negative							–	0.311^∗∗^

*^∗^*p* < 0.05 and ^∗∗^*p* < 0.01.*

### Testing of the Models in Study 1

**FIGURE 2:**
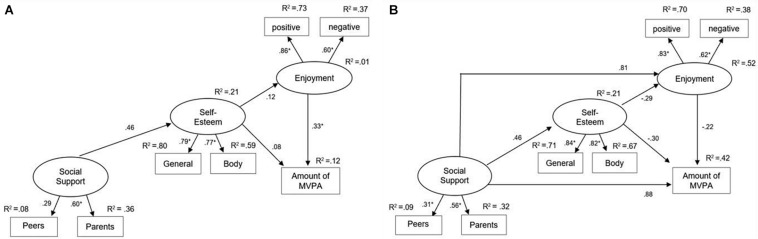
Original **(A)** and adapted model **(B)** for Study 1. Standardized regression weights are presented next to the arrows with ^∗^*p* < 0.05.

**FIGURE 3:**
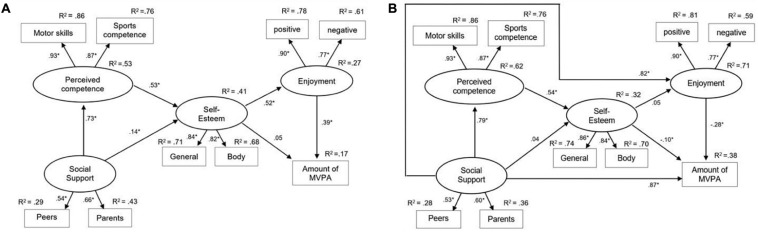
Original **(A)** and adapted model **(B)** for Study 2. Standardized regression weights are presented next to the arrows with ^∗^*p* < 0.05.

The fit indices for the original and the adapted model are presented in Table 4 with path coefficients in Figures 2, 3. The chi-square statistic for the original model significantly deviated from zero (χ*^2^* = 38.7; *df* = 11; *p* < 0.01) and CFI (0.87) and RMSEA (0.12) showed a poor model fit. Therefore, it can be concluded that the Weiss-Harter-model does not fit well to the data of Study 1.

**TABLE 4:** Model fit indices.

	**χ^2^**	***df***	***p***	**CFI**	**RMSEA**	**Δχ^2^**	**Δ *df***	***p***
**Pilot study** **Study 1**								
Model 1 (Original)	38.7	11	0.00	0.870	0.118			
Model 2 (Adapted)	4.8	9	0.85	1.000	0.000	33.9	2	<0.01
**Study 2**								
Model 3 (Original)	1112.6	22	0.00	0.871	0.148			
Model 4 (Adapted)	103.7	20	0.00	0.990	0.043	1008.9	2	<0.01

*df = degrees of freedom; p = probability value; CFI = Comparative Fit Index; RMSEA = Root Mean Square Error of Approximation; Δχ2 = chi-square difference; Δ df = difference of the degrees of freedom.*

In the adapted model, the direct paths between social support to PA enjoyment and social support to the amount of MVPA were added. By adding these two paths, the model fit improved significantly (Δχ*^2^* = 33.9; Δ*df* = 2; *p* < 0.01). The chi-square statistic did not deviate from zero (χ*^2^* = 4.8; *df* = 9; *p* ≥ 0.05) and CFI (1.00) and RMSEA (0.00) indicated an almost perfect model fit. Social support had a strong, but not significant effect on PA enjoyment (β = 0.81, *p* > 0.05) and PA (β = 0.88, *p* > 0.05). The amount of explained variance in PA was 42%. The effects of PA enjoyment and self-esteem on PA inverted the algebraic sign indicating negative non-significant effects on PA with medium effect sizes. The results of this study support the validity of the adapted model.

### Validating the Models in Study 2

In Study 2, both the original model and the adapted model were tested again. The construct perceived competence added to the models. The fit indices for the original and the adapted model are presented in Table 4. With regard to the original model, the chi-square statistic significantly deviated from zero (χ*^2^* = 1112.6; *df* = 22; *p* < 0.01) and CFI (0.87) and RMSEA (0.15) showed a poor model fit. Therefore, the results of Study 2 do not support the validity of the Weiss-Harter-model.

In the adapted model, both effects of social support on PA enjoyment and PA were added. The adapted model showed again a significantly improved model fit (Δχ*^2^* = 1008.9; Δ *df* = 2; *p* < 0.01). The chi-square statistic of the adapted model significantly deviated from zero (χ*^2^* = 103.7; *df* = 20; *p* < 0.01) but CFI (0.99) and RMSEA (0.04) indicated a very good model fit. Social support had strong, and significant effects on PA enjoyment (β = 0.82, *p* < 0.01) and PA (β = 0.87, *p* < 0.01). The associations between social support and perceived competence as well as perceived competence and self-esteem stayed the same. The other path coefficients were impacted by the addition of the new paths. The effect of social support on self-esteem became small to meaningless and non-significant (β = 0.04, *p* > 0.05) as well as the relationship between self-esteem and enjoyment (β = 0.05, *p* > 0.05). The algebraic signs of the effects of self-esteem and PA enjoyment on PA inverted again show negative significant effects. The amount of explained variance in PA was 38%. Therefore, the results of Study 2 support the validity of the adapted model.

## Discussion

The main purpose of this work was to examine a model of PA for children and adolescents proposed by Weiss (2000). The model proposes that self-esteem and PA enjoyment are the main determinants of PA whereas perceived competence and social support are the main determinants of self-esteem. In order to test this model, two studies have been conducted. In Study 1, the original model was tested and adapted. In Study 2, the procedure of testing the original and adapted model was applied again to validate the results of Study 1.

The results of both studies suggest that the original model does not fit the data. The original model proposes that self-esteem is the central predictor of PA behavior. Self-esteem is supposed to mediate the effects of perceived competence and social support on PA, and to be a central predictor of PA enjoyment. However, both studies do not provide evidence for such a central role of self-esteem in the process of motivating adolescents’ PA. In Study 1, self-esteem was not significantly related to PA and PA enjoyment neither in the original nor in the adapted model. In Study 2, the effects of self-esteem on PA were very small in both model-versions. To our knowledge, there are no other studies that apply the original Weiss-Harter-model to youth’s PA, thus we could only compare these studies’ findings to studies that investigated the relationship between PA and single components of the model.

With regard to the core variable self-esteem, studies have already shown its limited explanatory value for adolescent PA. For example, in a study with adolescents aged 11–15 years, the percentage of active students with low general self-esteem was not significantly different from students with high self-esteem (Inchley et al., 2011). In the same study, students with a higher physical self-worth were more active (Inchley et al., 2011). Another study with 11–14 year old adolescents showed that physical self-worth only had a small effect on PA (Raudsepp et al., 2002). Both studies considered students’ physical self-concept, but did not include social support in their analysis. Another study analyzed both effects of self-esteem and tangible/intangible parental social support on sports participation in students between 16 and 18 years. Both types of parental social support had the strongest direct effects on sports participation, while self-esteem did not have any direct effect but was rather mediated through social support (Qurban et al., 2019).

Thus, current evidence does not support a clear association between self-esteem and PA in youth and more research is needed, particularly using longitudinal and intervention study designs.

Based on current evidence, we hypothesized that social support might be an important determinant of PA and PA enjoyment, and thus tested an adapted model with direct connections between social support, PA and PA enjoyment. In doing so, the effect of self-esteem on PA enjoyment, which was substantial in the original model, disappeared. This may indicate that the effects of self-esteem on PA enjoyment are predominantly produced by social support. The adaptation of the model significantly improved the model fit in both studies providing robust replication evidence that the original model lacked the suggested paths. The effects of social support were strong for both PA and PA enjoyment. The amount of explained variance in PA as well as in PA enjoyment increased substantially when the effects of social support were included into the model. These findings are in line with published research that investigated the relationship between social support, PA enjoyment and adolescent PA. Substantial empirical evidence suggests that social support of peers and parents is a strong predictor of PA in children and adolescents (Sallis et al., 2000; Van Sluijs et al., 2007; Craggs et al., 2011; Fitzgerald et al., 2012; Geller et al., 2013; Mendonca et al., 2014), and in different settings such as leisure-time and school-PA and active commuting (Hohepa et al., 2007; Sabiston and Crocker, 2008). While PA during adolescence generally decreases, both parental and peer social support mitigate the decline (Davison and Jago, 2009; Dishman et al., 2017). More recently, it has also been shown that social support is a determinant of PA enjoyment in adolescents and young adults (Wienke and Jekauc, 2016; Leisterer and Jekauc, 2019a, b). In this context, the role of PA enjoyment has also been investigated in different variations. A study with 15 year-old adolescents showed that parental and peer social support have a direct effect on MVPA and on PA enjoyment (Silva et al., 2014). Two other studies showed the importance of intangible and tangible parental social support as well as the beliefs about parental social support for PA enjoyment, with the latter one predicting PA (Wing et al., 2016; Shen et al., 2018). These findings were also supported in two qualitative studies with adolescents, reporting that the interaction with peers leads to positive emotions during PE lessons (Leisterer and Jekauc, 2019b) and that social support influences PA through improving PA enjoyment and motivation as well as enabling PA (Laird et al., 2018).

In summary, the results from our current study as well as previously published research strongly suggest that social support by family and peers is an important determinant to influence PA behavior. Exercising in a group of supportive persons might mobilize the motivational resources for PA. Although social support was a strong predictor of PA enjoyment, the effects of social support on PA were mainly direct and only slightly mediated by PA enjoyment. These findings highlight the importance of social support as predictor of PA enjoyment and PA of adolescents.

However, the mechanisms of the indirect effects of social support are less clear (Jekauc et al., 2018). In both studies, the effects of PA enjoyment and self-esteem on PA inverted the algebraic sign suggesting that the effects of both variables were negative after including the effects of social support on PA enjoyment and PA. These counterintuitive results suggest that social support, self-esteem and enjoyment confound each other when influencing PA. We suppose that suppression effects might cause the inversion of the algebraic sign. As social support, self-esteem und PA enjoyment overlap in their variance when explaining PA behavior, some parts of the variance of self-esteem and PA enjoyment might be suppressed by social support. The remaining unsuppressed parts of the variance in self-esteem and PA enjoyment might have counter effects causing the inversion of the algebraic sign of the effects which means that social support possibly takes over the majority of the positive variance which then, practically speaking, only leaves active adolescents that do not enjoy PA and have low self-esteem. However, further studies are needed to examine this inversion of effects in more detail.

Perceived competence was assessed only in Study 2. Surprisingly, the inclusion of perceived competence into the model did not significantly alter the structure of the model. Perceived competence was significantly affected by social support and was a significant predictor of self-esteem. The effects of perceived competence on PA and PA enjoyment were rather indirect via self-esteem. However, empirical evidence suggests that perceived competence is a strong predictor of PA behavior (e.g., Sallis et al., 2000; Craggs et al., 2011) and PA enjoyment (Carroll and Loumidis, 2001; Cairney et al., 2012; Leisterer and Jekauc, 2019a, b). It is possible that the measure of self-competence in our questionnaire on physical self-concept might not be specific enough or appropriate. Further studies are needed to explain whether methodological or theoretical issues are responsible for these deviations in our findings from the literature.

### Strengths and Limitations

This study has several limitations, including the small sample size and the lack of measuring perceived competence in Study 1, which limited full comparability of the studies. Also, we used only subjective albeit validated measurement of PA, and the measurement of perceived competence might not have been specific enough for the PA domain to detect potential relationships between determinants. Another limitation pertains to the cross-sectional samples in both studies not allowing for causal inferences, requiring further longitudinal investigations to allow for more rigorous conclusions about temporality and causal mechanisms.

These limitations notwithstanding, the study has also several strengths. Using the procedure of cross-validation, this study provides robust results tested on two independent samples. Another strength is the theory-driven approach to improve our understanding of the complex relationship between various determinants of PA. We applied established measurement scales to investigate the complex relationship and used multivariate analyses to examine the structure of PA determinants in adolescents. Furthermore, the sample size of Study 2 is large enough to detect even small effects, and, due to its representative character, these results can be generalized for adolescents in Germany.

### Implications

Based on our findings, implications for future research and PA interventions can be outlined. The interplay between social support and PA enjoyment and the underling mechanisms are largely unknown. Therefore, further studies are needed to examine the structure of relationship between these constructs. Additionally, specific measurements of perceived competence related to PA should be developed or identified. Future studies should also test the model using samples across different countries and cultures, ideally by collecting longitudinal data that allows testing of the hypothesized longitudinal theoretical variable relationships.

With regard to the practical application of our study, interventions to increase PA behavior in adolescents should include components increasing social support by family and peers. When including parents in adolescent’s PA interventions, interventionists should ensure that parents are supporting their child without pressuring or controlling PA as these aspects have both been associated with less PA enjoyment, thereby leading to less PA (Amado et al., 2015; Wing et al., 2016). In terms of peer support, interventionist should consider the mutual relationship between peer’s PA levels as youth tend to befriend with peers who have similar activity levels (De La Haye et al., 2011). While this might be helpful for the active adolescents, one may conclude that inactive youth also befriend inactive peers, reinforcing inactivity. Therefore, interventions should target group building and friendship between active and inactive adolescents. Peer-delivered interventions could be a suitable approach to achieve this aim. While peer-delivered interventions have been neglected so far, they have shown to be as effective as professionally delivered interventions (Ginis et al., 2013). Taking peer-effects on PA enjoyment and motivation into account, this approach is worth consideration for future adolescent PA interventions.

### Conclusion

The results of both studies suggest that the original model proposed by Weiss (2000) needs to be adapted for youth. The prominent role of self-esteem in this model was not confirmed. Rather, the results imply that social support might have direct effects on PA and PA enjoyment. Future studies examining the interplay between social support, PA enjoyment and potentially perceived competence as determinants of PA are needed. Given the prominent role of social support in our studies, interventions to increase PA levels should include components of social support for adolescents.

## Data Availability Statement

Data cannot be shared publicly because of strict ethical conditions with which study investigators are obliged to comply: The Charitè – Universitätsmedizin Berlin ethics committee, the Karlsruhe Institute of Technology (KIT) ethics committee, and the Federal Office for the Protection of Data explicitly forbid making the data publicly available because informed consent from study participants did not cover public deposition of data. However, the minimal data set underlying the findings is archived at the Institute of Sports and Sports Science of the Karlsruhe Institute of Technology and can be accessed by interested researchers on site. On-site access should be submitted to the Institute of Sports and Sports Science, Karlsruhe Institute of Technology, Karlsruhe, Germany (info@sport.kit.edu).

## Ethics Statement

The studies involving human participants were reviewed and approved by the Charitè - Universitätsmedizin Berlin Federal Office for the Protection of Data. Written informed consent to participate in this study was provided by the participants’ legal guardian/next of kin.

## Author Contributions

DJ and CM wrote the manuscript and conducted the statistical calculations. AW and DJ conceptualized both studies. CN organized the data collection. CN, KW, CRN, and JK-R helped to conceptualize and edited the manuscript.

## Conflict of Interest

The authors declare that the research was conducted in the absence of any commercial or financial relationships that could be construed as a potential conflict of interest.
